# Supplementary material for: The Effects of COVID-19 on Placenta and Pregnancy: What Do We Know So Far?
Source: Diagnostics (Basel). 2021 Jan 8;11(1):94. doi: 10.3390/diagnostics11010094 (PMC7827584; doi:10.3390/diagnostics11010094)
Supplement: Supplementary file 1 [file diagnostics-11-00094-s001.pdf]

diagnostics

Review

The Effects of COVID-19 on Placenta and Pregnancy: What Do We Know So Far?

Yin Ping Wong [1,*], Teck Yee Khong [2] and Geok Chin Tan [1,*]

1 Department of Pathology, Faculty of Medicine, Universiti Kebangsaan Malaysia, Jalan Yaacob Latif, Bandar Tun Razak, 56000 Kuala Lumpur, Malaysia
2 Department of Pathology, SA Pathology, Women's and Children's Hospital, North Adelaide SA 5006, Australia; yee.khong@adelaide.edu.au
* Correspondence: ypwong@ppukm.ukm.edu.my (Y.P.W.); tangc@ppukm.edu.my (G.C.T.);
Tel.: +60-391-455-364 (Y.P.W); +60-391-455-362 (G.C.T)

Table 1. Summary of Reports on Clinical Characteristics of SARS-CoV-2 included Studies.

Authors	Number of Cases	Clinical History	Mum tested COVID-19 Positive	Placenta Sent for SARS-CoV-2	Placental Morphological Features	IHC/ISH Findings	Ultrastructural Features	Fetal Outcomes *
** Algarroba et al. 2020 [16]	1	40y/o G3P2, 28 + 4wks' gestation with sepsis pneumonia secondary to severe COVID-19. Delivered via CS	6 days prior delivery	RT-PCR, IHC, TEM (All +ve)	Gross: 271 g (75–90th percentile) Histo: mature chorionic villi with focal villous oedema, an area of decidual vasculopathy on maternal surface	(IHC) +ve SARS-CoV-2 glycoprotein on placental cells	Virions in syncytiotrophoblast, microvillus and cell process of fibroblasts	BW: 1340 g. Neonate tested -ve
Algeri et al. 2020 [17]	5	27 + 4 to 38 + 4wks' gestation, 2 out of 5 with severe COVID-19 Delivered via 4CS, 1VD	3 cases +ve on admission, 2 cases +ve PP	Not tested	One placenta (38 + 4wk' gestation) sent for histology. Histo: no sign of infection/inflammation	NA	NA	BW: NA. All neonates tested -ve
Baergen and Heller 2020 [18]	20 (1 twins)	16–41y/o, 32 + 2–40 + 4wks' gestation.Delivered via 5CS, 15VD	All +ve, Unclear when	Not tested	Signs of MVM: infarction (n = 2), accelerated villous maturation (n = 4), intervillous thrombosis (n = 1). Signs of FVM: thrombi in fetal circulation (n = 3), intramural fibrin (n = 7), chorangiosis (n = 1), VSK (n = 3), avascular villi (n = 3) Inflammation: chorioamnionitis (n = 1), chronic villitis (n = 4), fetal vasculitis (n = 2). Others: meconium (n = 7), cord abnormalities (n = 2), chorionic cysts (n = 1)	NA	NA	BW: 2072–4115 g, SGA (n = 2) All neonates tested -ve

** Baud et al. 2020 [19]	1	28y/o G1P0, 19wks gestation, with fever in imminent labour. Delivered via VD	2 days prior delivery	RT-PCR (+ve)	Neutrophils and monocytes in the subchorial space, increased intervillous fibrin deposition, and funisitis.	NA	NA	Stillbirth, BW: NA. Autopsy: no malformation, Neonate, cord blood and AF tested –ve
Chen S et al. 2020 [20]	3	23, 32 and 34y/o, 35–38 + 6wks' gestation.All delivered via CS	1, 2 and 4 days PP	RT-PCR (-ve)	Increased intervillous and/or subchorionic fibrin deposition (*n* = 3)<br>Increased in villous syncytial nodules (*n* = 2)<br>Massive infarction (*n* = 1)<br>Chorionic haemangioma (*n* = 1)	NA	NA	Umbilical cords and neonates all tested -ve
Chen Siyu et al. 2020 [21]	5	25–31y/o, 38 + 4–40 + 4wks' gestation	0, 1 and 3 days PP	Not tested	No significant pathology (No placental infarction and chorionic amniotic inflammation)	NA	NA	BW: 3235–4050 g. All neonates tested -ve
** Facchetti et al. 2020 [22]	15	26–44y/o, 32 + 6–41 + 3wks' gestation	All +ve, Unclear when	IHC, TEM (only 1 +ve)	Signs of MVM: infarction (*n* = 7), increased perivillous fibrin deposition (*n* = 4), accelerated villous maturation (*n* = 5), decidual vasculopathy (*n* = 2), intervillous thrombosis (*n* = 4), subchorial haematoma (*n* = 1) Signs of FVM: Chorangiosis (*n* = 4), VSK (*n* = 1), avascular villi (*n* = 1). Inflammation: chorioamnionitis (*n* = 1), villitis (*n* = 1), intervillositis (*n* = 2), acute deciduitis (*n* = 2), choriovasculitis &/ fetal vasculitis (*n* = 2)<br>No significant pathology (*n* = 1)	(IHC) +ve SARS-CoV-2 S & N proteins in STs. Monocytes-macrophages & neutrophils (CD11c, CD14, CD68, CD163). Scattered CD3+-lymphocytes. PD-L1 weak-moderate +ve	SARS-CoV-2 in villous syncytiotrophoblast, Hofbauer cells, fetal endothelial capillary cells & intravascular mononuclear cells, fibroblasts, in maternal macrophages	BW: 1499–4150 g. One neonate tested +ve
** Ferraiolo et al. 2020 [23]	1	30y/o, G1P0, 38 + 3wks' gestation, asymptomatic. Delivered via CS for breech presentation	+ve PP	RT-PCR (+ve)	Gross: Mild subchorionic fibrin deposition & a single ischaemic area in chorionic disc, slightly hyperspiralised umbilical cord. Histo: signs of villous delayed maturation, focal microchorangiosis,moderate fibrin deposition, villous agglutination, intervillous thrombosis	NA	NA	BW: NA<br>Neonate tested -ve

Gulersen et al. 2020 [24]	50	24.25–33.75y/o, 38.45–40.1wks' gestation, with COVID-19 symptoms: 9 mild, 3 moderate, 2 severe, 1 critical disease. Delivered via 12CS, 38VD	0–20 days prior delivery	Not tested	Signs of MVM: infarction (*n* = 4), distal villous hypoplasia (*n* = 2), increased perivillous fibrin (*n* = 6), intervillous thrombosis (*n* = 13), retroplacental thrombus (*n* = 2), old haemorrhage in membranes (*n* = 1), Signs of FVM (*n* = 4), delayed villous maturation (*n* = 10), chorangiosis (*n* = 3). Inflammation: chorioamnionitis (*n* = 9), villitis (*n* = 2), chorionitis (*n* = 11), fetal vasculitis (*n* = 7). Others: meconium (*n* = 9)	NA	NA	BW: 2771–3435 g All neonates tested -ve
** Hecht et al. 2020 [25]	20	22–42y/o, 22–41+1wks' gestation, 2 sets of twins, 7 asymptomatic, 8 mild symptoms, 5 severe COVID-19. Delivered via 12CS, 8VD	9 days prior delivery to 1 day PP	IHC & ISH (3 +ve)	Placental weight: 54–797 g Signs of MVM: infarction (*n* = 2), increased perivillous fibrin deposition (*n* = 2), decidual vasculopathy (*n* = 3), intervillous / subchorionic thrombosis (*n* = 4) Signs of FVM: Chorangiosis (*n* = 1), VSK (*n* = 1), avascular villi (*n* = 2). Inflammation: chorioamnionitis/subchorionitis (*n* = 7), villitis (*n* = 1), intervillositis (*n* = 1), chronic deciduitis (*n* = 2), choriovasculitis / fetal vasculitis (*n* = 4). Others: meconium (*n* = 5), villous oedema (*n* = 2), hypercoiling cord (*n* = 1), marginal insertion of cord (*n* = 1)	(IHC) +ve SARS-CoV-2 on ST (*n* = 2). (ISH) +ve SARS-CoV-2 on STs, CTs & decidual endothelial cells (*n* = 3). +ve ACE2 on STs, CTs and EVTs. TMPSSR2 weak in villous endothelium & rarely in STs (*n* = 10)	NA	BW: NA One IUD (not tested), One neonate tested +ve
** Hosier et al. 2020 [26]	1	35y/o, G3P1, 22wks' gestation.	+ve on admission	RT-PCR (+ve)	Gross: marginal adherent blood clot associated with a focal placental infarct. Histo: diffuse perivillous fibrin & histiocytic intervillositis.	(IHC & ISH) +ve SARS–CoV-2 S protein in STs	+ve virus particles within the cytosol of placental cells	Umbilical cord tested +ve & Neonate tested -ve. Autopsy: unremarkable
** Hsu et al. 2020 [27]	1	29y/o, multigravida, 40+4wks' gestation, with mild COVID-19 disease. Delivered via VD	2 days prior delivery	IHC (+ve)	Gross: marginally inserted umbilical cord, 538g (60th percentile). Histo: hypertrophic arteriolopathy & subchorionic	(IHC) +ve SARS-CoV-2 N protein in chorionic villi endothelial cells, rarely trophoblasts	NA	BW: 3561g Neonate tested -ve

					laminar necrosis, chronic villitis, scattered islands of EVT			
** Kirstman et al. 2020 [28]	1	40y/o, G2P1, 35+5wks' gestation. Delivered by CS	1 day prior delivery	RT-PCR (+ve)	Chronic histiocytic intervillositis & extensive early infarction	CD68+ macrophages, CD3+ T cells, CD20+ B cells identified.	NA	BW: 2930 g Neonate tested +ve
Kuhrt et al. 2020 [29]	1 (twins)	30y/o, G2P0+1, monochorionic diamniotic (MCDA) twin, 32+6wks' gestation.Delivered by CS	2 days prior delivery	Not tested	Accelerated villous maturation & mild hypoperfusion	NA	NA	BW: 2190 g & 2160 g Both neonates tested -ve
Lokken et al. 2020 [30]	46 (only 1 examined)	38y/o, G6P2, 38+5wks' gestation. Delivered by VD	+ve on admission	RT-PCR (-ve)	SGA placenta (<5%), acute chorioamnionitis, mild funisitis, severe chronic villitis	IHC for cytomegalovirus -ve	NA	Autopsy: no abnormalities Neonate tested -ve
** Menter et al. 2020 [31]	5	27.6–39.7y/o, 39–40+5wk, with mild COVID-19 (*n* = 3), asymptomatic (*n* = 2). Delivered via 2CS, 3VD,	4, 5, 18, 35 days prior delivery & 1 day PP	RT-PCR & ISH (+ve in one case)	Placenta weight: 526–649 g Signs of MVM: infarction (*n* = 2), increased perivillous/intervillous fibrin deposition (*n* = 6), decidual vasculopathy (*n* = 3), accelerated villous maturation (*n* = 1), intervillous thrombosis (*n* = 1) Signs of FVM: thrombi in fetal circulation (*n* = 2), delayed villous maturation (*n* = 1), chorangiosis (*n* = 2). Inflammation: chronic villitis (*n* = 2), chronic histiocytic intervillositis (*n* = 1), chorioamnionitis/subchorionitis (*n* = 3), chronic deciduitis (*n* = 2), choriovasculitis / fetal vasculitis (*n* = 3). Others: meconium (*n* = 1), hypercoiling cord (*n* = 3), marginal insertion of cord (*n* = 1), placenta accreta (*n* = 1)	(ISH) +ve SARS CoV-2 RNA in endothelial cells of decidua. (IHC) weak +ve ACE2 in invasive EVT (*n* = 4). CD8+ T cells, CD68+ macrophages	NA	BW: 2790–3500 g, Umbilical cord blood, breast milk, amniotic fluid tested -ve No data on neonates
** Mongula et al. 2020 [32]	1	27y/o G2P1 @ 32+1wks' gestation.Delivered via CS	+ve on admission	RT-PCR (+ve)	Placenta grossly normal. Histo: increase in perivillous fibrin (>30–40%) leading to trophoblast necrosis, reduced intervillous space possible infarction, extensive histiocytic & neutrophilic intervillositis	(IHC) +ve SARS-CoV-2 in trophoblasts & stromal cells	NA	BW: 1920 g (58th percentile) Neonate tested -ve

Mulvey et al. 2020 [33]	5	26–40y/o, 38–40wks' gestation, with fever (*n* = 2), others asymptomatic (*n* = 3) Delivered 4VD, 1 CS	All +ve intrapartum	IHC & ISH (-ve)	Signs of FVM: thrombosis in larger vessels in the fetal circulation (*n* = 5), intramural fibrin (*n* = 4), VSK (*n* = 1), chorangiosis (*n* = 1), avascular villi (*n* = 1), Signs of MVM: perivillous fibrin (*n* = 1), others: furcate insertion of cord (*n* = 1), meconium (*n* = 3)	(IHC) -ve SARS-CoV-2 S protein & (ISH) -ve viral RNA in placenta +ve Complement staining C3d, C4d, C5b-9 in decidua, villi & perivillous fibrin (no different with controls)	NA	No data on neonatal outcome
Ozer et al. 2020 [34]	1	37y/o, G2P1, 39wks' gestation, with mild COVID-19 symptoms	+ve on admission	Not tested	Gross: 18 × 17 × 2.5cm, 564g (75th percentile), subchorionic fibrin on fetal surface. Histo: low grade villitis of unknown etiology, chronic deciduitis & avascular villi adjacent to a subchorionic thrombosis	(IHC) CD163+ macrophages, CD4+ lymphocytes,	NA	BW: 3360 g Neonate tested -ve
** Patane et al. 2020 [35]	22 (only 2 examined)	35.1 & 37.6wks of gestation, with fever & cough. Delivered by CS (for nonreassuring fetal status), VD	+ve intrapartum	ISH (2 +ve)	Chronic histiocytic intervillositis	(ISH) both +ve SARS-CoV-2 S protein in STs, (IHC) CD68+ macrophages	NA	BW: 2686 g, 2660 g One neonate tested +ve (at birth)
Prabhu et al. 2020 [36]	70 (only 29 examined)	26.1–37.2y/o, 37.4–39.9wks' gestation, COVID-19 symptoms (*n* = 15). Delivered via 32CS, 38VD	+ve on admission	Not tested	Signs of MVM (*n* = 8), Signs of FVM (*n* = 14), Inflammation: chronic villitis (*n* = 5), maternal response (*n* = 1), maternal & fetal response (*n* = 2), Others: meconium (*n* = 18), cord abnormalities (*n* = 1)	NA	NA	BW: 3211.08 g (mean) 1 stillbirth. All neonates tested -ve
** Pulinx et al. 2020 [37]	1 (twins)	30y/o, G2P1, 24wks' gestation, DCDA twin.. Delivered via VD	2 wks prior delivery	RT-PCR (+ve) & IHC (+ve)	Extensive intervillous fibrin deposition, ischaemic necrosis of surrounding villi, chronic histiocytic intervillositis, nuclear debris within fetal circulation & increased in erythroblasts	(IHC) +ve SARS-CoV-2 in STs (IHC) CD3+, CD8+ T lymphocytes, CD68+ macrophages	NA	1 IUD, 1 prepartal death, AF tested +ve, amniotic sac tested -ve, Neonates not tested
** Richtmann et al. 2020 [38]	5	24–40y/o, 21+1 to 38+3wks' gestation, with mild to moderate COVID-19 diseases. Delivered via 2CS, 3VD	+ve from 1 -22 days prior delivery	RT-PCR (+ve in 2 cases)	Signs of MVM: Increased in intervillous fibrin (*n* = 2), subchorionic thrombosis (*n* = 1), Inflammation: chorioamnionitis (*n* = 5), acute villitis (*n* = 2), acute intervillositis (*n* = 2), acute deciduitis (*n* = 2)	NA	NA	All 5 stillbirths. BW: 329–2895 g Autopsy (one case): no abnormalities, Neonates not tested for SARS-CoV-2

								AF +ve (in one case)
Shanes et al. 2020 [39]	16	23–41y/o, 16-40wks' gestation. Delivery method: NA	0, 1, 2, 7, 25, 28, 34 days prior delivery	Not tested	Placenta weight: 298–612 g Signs of MVM: Infarction (*n* = 4), accelerated villous maturation (*n* = 2), increased fibrin deposition (*n* = 3), intervillous thrombosis (*n* = 6), villous agglutination (*n* = 3), decidual vasculopathy (*n* = 7), retroplacental haematoma (*n* = 1). Signs of FVM: intramural fibrin (*n* = 1), avascular villi (*n* = 4), delayed villous maturation (*n* = 4), chorangiosis (*n* = 4). Inflammation: chorioamnionitis (*n* = 1), fetal vasculitis (*n* = 1), chronic deciduitis (*n* = 2), chronic villitis (*n* = 2), Others: hypercoiling of cord (*n* = 1), accreta (*n* = 2), villous oedema (*n* = 4), nucleated red cells (*n* = 1)	NA	NA	1 IUD, 5 SGA, 1 LGA All neonates tested -ve
** Sisman et al. 2020 [40]	1	37y/o, G4P3, 34wks' gestation. Delivered via VD	3 days prior delivery	IHC & TEM (+ve)	Large for gestational age, patchy chronic histiocytic intervillositis, villitis associated with VSK & necrosis, focal basal chronic vilitis, focal parabasal infarct, & features of meconium exposure in the fetal membranes	(IHC) +ve SARS-CoV-2 N protein in STs (IHC) CD68+ve macrophages	89–129 nm diameter structures, consistent with viral particles, clustered within membrane bound cisternal spaces in the STs	BW: 3280 g, Neonate tested +ve
Smithgall et al. 2020 [41]	51	19–47y/o, < 37wks' gestation (*n* = 10), ≥37wks' gestation (*n* = 41), majority had mild COVID-19 symptoms, 4 had severe disease. Delivered via 25CS, 26VD	+ve on admission	IHC & ISH (-ve)	Signs of MVM: Infarction (*n* = 7), accelerated villous maturation (*n* = 10), villous agglutination (*n* = 21), decidual vasculopathy (*n* = 3), intervillous thrombosis (*n* = 8), subchorionic thrombosis (*n* = 9), Signs of FVM: thrombi in fetal circulation (*n* = 4), avascular villi (*n* = 5), chorangiosis (*n* = 8). Inflammation: maternal response (*n* = 17), fetal response (*n* = 9),	(IHC) -ve SARS-CoV-2 S protein & (ISH) -ve viral S protein in placenta	NA	All neonates tested -ve

					chronic villitis (*n* = 2), No significant different from controls except villous agglutination & subchorionic thrombi			
** Taglauer et al. 2020 [42]	15	31.8y/o (mean age), 38.1wks' mean gestation age, no further clinical data	+ve intrapartum	IHC & ISH (all +ve)	Placental weight: 323–794 g Signs of MVM: Infarction (*n* = 5), increased fibrin deposition (*n* = 7), hypoplastic villi (*n* = 1), Inflammation: chorioamnionitis (*n* = 2), subchorionitis (*n* = 5), chronic villitis (*n* = 1), fetal choriovasculitis (*n* = 1), Others: meconium (*n* = 8), hypercoiling of cord (*n* = 3). No significant different from controls	(IHC & ISH) +ve SARS-CoV-2 S protein in STs & intermittent in CTs (ISH) ACE2 +ve in STs & TMPRSS2 -ve	NA	BW: 3319.9 g (mean) 5 neonates tested +ve (< 24 hours)
**Vivanti et al. 2020 [43]	1	23 y/o, G1P0, 35+2wks' gestation, with fever and severe cough & expectoration	3 days prior delivery	RT-PCR & IHC (+ve)	Gross: Perivillous fibrin deposition with infarction, as irregular strands of pale yellow-white induration. Histo: Diffuse perivillous fibrin deposition with infarction & acute and chronic intervillositis	(IHC) +ve SARS-CoV-2 N protein in perivillous trophoblasts (IHC) -ve Parvovirus & CMV	NA	BW: 2540 g, AF & neonate tested +ve, complicated with neurological deficit
** Zhang et al. 2020 [44]	74	38–40wks' gestation.Delivered via 20CS, 54VD	Likely upon admission	ISH (+ve in 2 cases of 53 placentas tested)	Signs of MVM: Infarction (*n* = 7), decidual vasculopathy (*n* = 36), retroplacental haemorrhage (*n* = 3), massive fibrin deposition (*n* = 2), Signs of FVM: thrombi in fetal circulation (*n* = 18), avascular villi (*n* = 5), Inflammation: chorioamnionitis (*n* = 48), villitis (*n* = 17), Others: meconium (*n* = 24), hypercoiling of cord (*n* = 3). No significant different from controls	(ISH) +ve SARS-CoV-2 in STs & endometrial glands (IHC) CD68+ macrophages, CD42b+ platelet aggregates	NA	One neonate tested +ve

* Neonate(s) COVID-19 status within 24 h of birth; ** Cases with SARS-CoV-2-infected/positive placentas (total cases, *n* = 36). Abbreviations: AF: amniotic fluid, BW: birth weight, CS: Caesarean section, CT: cytotrophoblasts, DCDA: dichorionic diamniotic, EVT: extravillous trophoblasts, FVM: fetal vascular malperfusion, G: gravida, histo: histopathology, IHC: immunohistochemistry, ISH: in situ hybridisation, IUD: intrauterine death, LGA: large for gestational age, MVM: maternal vascular malperfusion, N protein: nucleocapsid protein, NA: not available, -ve: negative, +ve: positive, P: para, PD-L1: programmed death-ligand 1, PP: postpartum, RNA: ribonucleic acid, RT-PCR: Reverse transcription-polymerase chain reaction, S protein: spike protein, SGA: small for gestational age, ST: syncytiotrophoblasts, TEM: transmission electron microscopy, VD: vaginal delivery, VSK: villous stromal-vascular karyorrhexis, wks: weeks, y/o: year-old.
